# Clinical features of depressive states in eating disorders

**DOI:** 10.1192/j.eurpsy.2024.1168

**Published:** 2024-08-27

**Authors:** A. Barkhatova, A. Smolnikova, S. Sorokin

**Affiliations:** Department of endogenous mental disorders and affective states, Mental health research center, Moscow, Russian Federation

## Abstract

**Introduction:**

Eating disorders (ED) are one of the most pressing problems of modern society. Eating disorders, due to their heterogeneity, can be considered both an independent form of mental disorders and as part of the manifestations of other mental illnesses. In the vast majority of cases within these pathologies, eating disorder coexists with depressive symptoms, which significantly worsens the prognosis of the disease.

**Objectives:**

Identification of the association of depressive disorders with eating disorders to improve the criteria for nosological diagnosis, prognosis and therapeutic approaches.

**Methods:**

A total of 74 patients aged from 15 to 25 years old (all female, average age 16.2), who were on outpatient and inpatient observation of the clinic were studied.

**Results:**

The study made it possible to establish the characteristics of depressive disorders and the nature of the current course of depression associated with eating disorders. In eating disorders with a predominance of **anorexia nervosa**, the structure of depression was more dominated by the asthenia radical with symptoms of apathy, melancholia, anhedonia, irritability, episodes of anxiety after eating, and sleep disturbances. Patients noted a decrease in performance, mental activity, and a narrowing of their range of interests and communication. Depression became severe as exhaustion progressed. For eating disorders with **bulimia nervosa**. depressive states varied in the severity and polymorphism of their manifestations. Their structure was largely dominated by the apatho-adynamic radical of affect, along with asthenia and anxiety, which often reached the level of panic states. Often, along with this, there were pronounced a guilt feeling and low self-esteem ideas with self-deprecation and self-hatred, which led to the manifestation of auto-aggressive behavior (both non-suicidal and suicidal). Depression reached a severe degree as exhaustion progressed, as well as against the background of more frequent attacks of overeating and vomiting.

**Conclusions:**

The identified associations between depressive disorders and eating disorders allow us to form a clearer picture of the expected course of psychiatric disease and optimize therapeutic intervention algorithms.

**Disclosure of Interest:**

None Declared

